# Image resizing using saliency strength map and seam carving for white blood cell analysis

**DOI:** 10.1186/1475-925X-9-54

**Published:** 2010-09-20

**Authors:** ByoungChul Ko, SeongHoon Kim, JaeYeal Nam

**Affiliations:** 1 Department of Computer Engineering, Keimyung University, Shindang-Dong, Dalseo-Gu, Daegu, Korea

## Abstract

**Background:**

A new image-resizing method using seam carving and a Saliency Strength Map (SSM) is proposed to preserve important contents, such as white blood cells included in blood cell images.

**Methods:**

To apply seam carving to cell images, a SSM is initially generated using a visual attention model and the structural properties of white blood cells are then used to create an energy map for seam carving. As a result, the energy map maximizes the energies of the white blood cells, while minimizing the energies of the red blood cells and background. Thus, the use of a SSM allows the proposed method to reduce the image size efficiently, while preserving the important white blood cells.

**Results:**

Experimental results using the PSNR (Peak Signal-to-Noise Ratio) and ROD (Ratio of Distortion) of blood cell images confirm that the proposed method is able to produce better resizing results than conventional methods, as the seam carving is performed based on an SSM and energy map.

**Conclusions:**

For further improvement, a faster medical image resizing method is currently being investigated to reduce the computation time, while maintaining the same image quality.

## Background

Peripheral blood cell differential counting provides valuable information for accurate patient diagnoses, yet the microscopic review is labor intensive and requires a highly trained expert. Current automated cell counters are based on laser-light scatter and flow-cytochemical principles, nonetheless, 21% of all processed blood samples still require microscopic review by experts [1]. Therefore, various efforts [1-5] have already been made to develop automatic cell analysis systems using image processing. Blood cell images consist of both white and red blood cells scattered across the entire image, however, it is the white blood cells (WBCs) that provide the important information for patient diagnoses, such as leukemia or cancer [2]. Thus, in most research, WBC segmentation is the important procedure, where the ultimate goal is to extract all the WBCs from a complicated background and then segment the WBCs into their morphological components, such as the nucleus and cytoplasm.

Representative WBC analysis systems, such as Cellarvision Diffmaster Octavia [4] and Cellarvision DM96 [5], scan the whole slide at a low magnification first to identify potential WBCs using the specific characteristics of WBCs, such as their color, size, and shape, and then take digital images at a high magnification. Thereafter, pre-classification is performed using only the cropped digital images. While this method is more efficient than scanning WBCs from a high-resolution image of the whole slide, additional time is required for the WBC search, especially when the image contains several WBCs. Furthermore, additional storage is needed to save the individual potential WBCs and extra time required to classify the WBCs, as the system has to check all potential WBC images to analyze just one slide.

Meanwhile, other methods [2,3] use only an original high-resolution image for the WBC analysis. However, analyzing WBCs from the whole image is time consuming, since the size of blood cell images is normally at least 800 × 600. Therefore, an image-resizing method is needed that retains all the WBCs without morphological distortion in order to reduce the post-segmentation classification time. Furthermore, since resized high-quality images require less storage, the post-image segmentation and classification can be more accurate than with conventional image compression, such as JPEG.

Related work can be divided into two parts; image compression and image resizing.

First, various lossless compression techniques already exist that can preserve the characteristics of an image, yet with a low compression rate. For example, several researchers [6-8] have proposed transform coding schemes, such as a Principal Component Analysis (PCA) and Discrete Cosine Transform (DCT), while Karras et al. [9] used a discrete wavelet transformation (DWT) and fuzzy c-means clustering technique. Plus, to achieve higher compression rates without detracting from the quality, region of interest (ROI) methods with a DCT have also been investigated [6,10]. In particular, Gokturk et al. [10] proposed a hybrid model, using lossless compression in regions of interest and high-rate motion-compensated lossy compression in other regions in the case of a sequence of CT images. Nonetheless, even though lossless compression produces a higher compression rate without distorting ROIs, the exact preservation of a ROI is still difficult when the compression rate is above a specific limitation. Therefore, a new algorithm is needed that can efficiently preserve ROIs, regardless of the compression rate.

**Figure 1:**
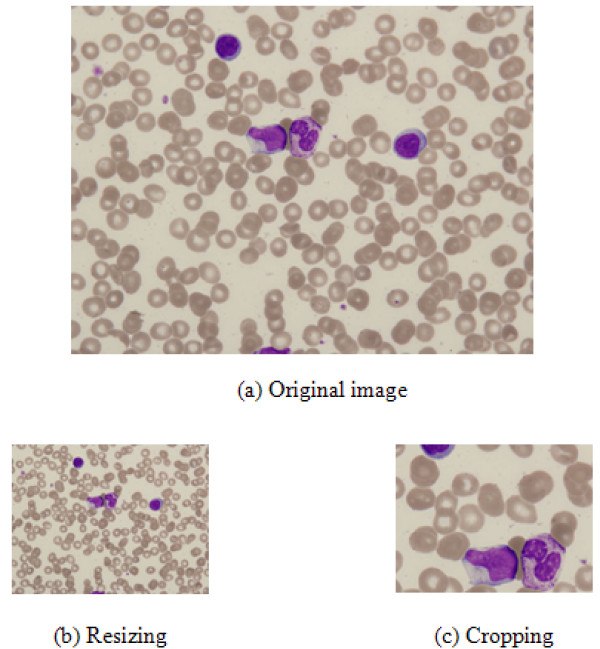
**Examples of different scaling-down methods**.

In addition to image compression methods that merely preserve the original image size, some researchers have attempted to resize or crop [11,12] images according to the image contents. Yet, as shown in Fig. 1-(b), standard resizing homogeneously reduces the image size, thereby damaging all the image contents based on the ratio of the resizing. Similarly, while cropping can be used to display the most important region in an image, as shown in Fig. 1-(c), cell images contain many ROIs, making cropping inappropriate for cell image compression.

**Figure 2:**
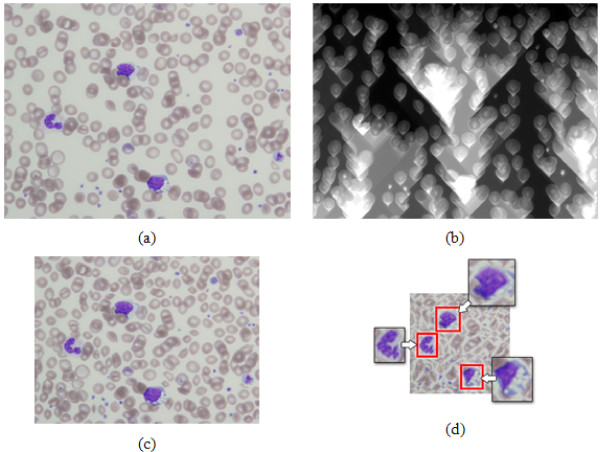
**Example of seam carving**: (a) original image, (b) energy map of (a), (c) reduced image using seam carving that preserves shape of important contents (WBCs), (d) distorted image using harsh seam carving, where shapes of WBC nuclei are distorted.

Meanwhile, seam carving [13] changes the size of an image by subtly removing or inserting a connected path of pixels from a different part of the image according to the measured energy, as shown in Fig. 2-(b). However, even though seam carving can efficiently remove non-ROI pixels, if the operation is applied too harshly (i.e. resizing an 800 × 600 image to 200 × 120), important ROIs can still be damaged, as shown in Fig. 2-(d). Furthermore, since seam carving was originally developed for nature images, its application to medical images is somewhat limited. For example, the energy distribution of WBCs is not distinctive in blood images, making it hard to apply a seam carving operator to blood cell images, which results in an inevitable removal or insertion of pixels in WBCs.

Accordingly, this paper presents a new method for resizing blood cell images while preserving the size and shape of WBCs. In peripheral blood, WBCs are divided into five classes according to their maturation stage, making it essential to preserve the size and shape of the nucleus. Thus, to provide an efficient image-resizing method that treats WBCs as ROIs, a Saliency Strength Map (SSM) is proposed using a visual attention model and the structural properties of WBCs to generate a new energy map. As such, this map maximizes the energies of the WBCs, while minimizing the energies of the red blood cells and background. Therefore, in contrast to previous algorithms, the main contribution of this study is to improve the resizing performance with a lower file size, while preserving the WBCs using the proposed SSM with an energy map.

**Figure 3:**
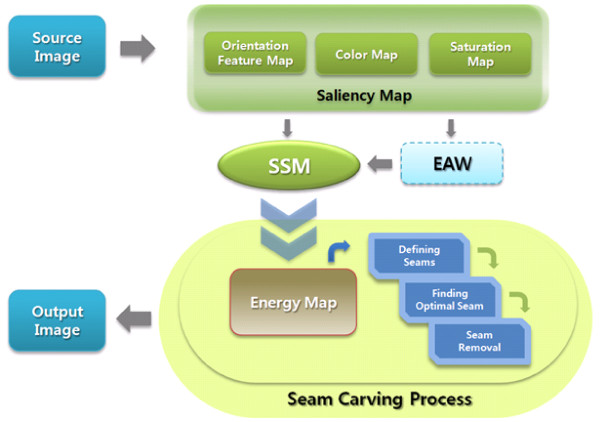
**Architecture of proposed method**.

Fig. 3 shows the architecture of the cell image resizing using the proposed SSM and seam carving.

The remainder of this paper is organized as follows. **Methods **describes the algorithms used to create the saliency strength map, an Ellipse Attention Window (EAW) that removes useless regions from the image, and the seam removal using an energy map based on the saliency strength. **Results and Discussion **evaluates the accuracy and applicability of the proposed resizing method based on experiments, and some final conclusions and areas for future work are presented in **Conclusions**.

## Methods

This paper proposes a new image-resizing method with a lower file size that can efficiently preserve WBCs using a visual saliency map based on the following two assumptions:

▪ the nuclei of WBCs are nearly round.

▪ the nuclei of WBCs are highlighted in purple on a white background with mono-chromatic red blood cells.

Using these characteristics, a Saliency Strength Map (SSM) is proposed using a visual attention model, while the structural properties of WBCs are used to generate an energy map.

### Saliency map generation

In contrast to nature images, microscopic images, especially blood cell images, have different characteristics with distinct diagnostic meanings, such as a varying color and saturation according to fluorescence staining. For example, in the case of blood cell images, the salient parts, the WBCs, tend to be highly saturated and purple in color, while the remaining parts, the red blood cells, have a more monotonous appearance. Thus, for semantic seam carving, knowledge of the exact positions of the relevant WBCs is crucial. Therefore, to obtain the position of WBCs, a modified visual attention model is used, as proposed in our previous research [14].

**Figure 4:**
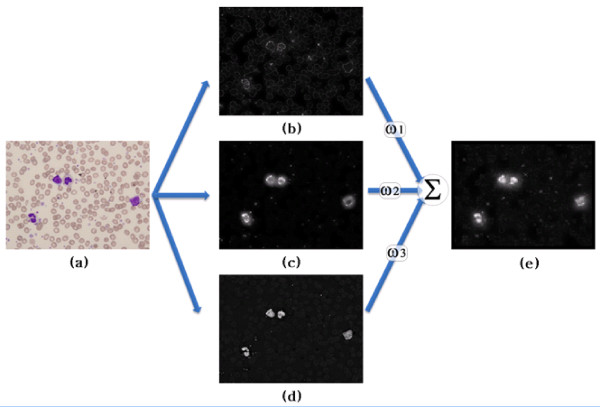
**Flow diagram of saliency map**: (a) source image, (b) orientation map, (c) color map, (d) saturation map, and (e) saliency map.

The original saliency-based visual attention model was proposed by Itti et al. [15], and uses color, luminance, and orientation. The most salient areas are then selected based on a winner-take-all competition map. However, in this study, a saliency map is used for the initial detection of Attention Windows based on a weighted linear combination of a color map, saturation map, and orientation map as shown in Fig. 4.

To produce the color map, this study uses a CIE Lab color model, where each a* and b* image is down-sampled to half the size of the original image. Different sized filters *s *∈ *{11 × 11,13 × 13} *are then applied to the down-sampled *a *and *b *images. The filters estimate the center-surround difference between the center point and the surrounding points within the filter scale *s *using two colors *c *∈ *{a, b}*, and this difference yields the feature map, *C(c, s)*. The size of the filters is typically chosen based on the number of available observations. In this study, the size of *s *was set at 11 × 11 and 15 × 15 based on several experiments using an 800 × 600 image size. Hence, the filter size can be changed according to the image size, with a half value when using a 400 × 300 image size and vice versa. In Eq. (1), the normalized color difference map $\bar{C}$ is estimated from *C(c, s)*s.

(1) $$\bar{C}=\frac{1}{4}(\sum_{c\in \{a,b\}}\sum_{s\in \{11\times 11,13\times 13\}}C(c,s))$$

In parallel with the color feature map, the orientation feature map is produced using a simple wavelet transform. After a one-level wavelet transform, horizontally (LH), vertically (HL), and diagonally (HH) orientated sub-images are obtained from the wavelet subbands. The orientation feature map, *O(c, s) *is then produced from the three sub-images *c *∈ *{HH, HL, LH} *and two filters *s *∈ *{11 × 11,13 × 13} *using the same method as for the color feature map. In Eq. (2), the normalized orientation difference map $\bar{O}$ is estimated from *O(c, s)*s.

(2) $$\bar{O}=\frac{1}{6}(\sum_{c\in \{HH,HL,LH\}}\sum_{s\in \{11\times 11,13\times 13\}}O(c,s))$$

The normalized saturation feature map $\bar{S}$ is processed in a similar way to the color map using a saturation feature map, *S(c, s) *from HIS color space and two filters *s *∈ *{11 × 11,13 × 13} *based on the following Eq.

(3) $$\bar{S}=\frac{1}{2}(\sum_{c\in \{s\}\;s\in \{11\times 11,13\times 13\}}S(c,s))$$

After the three feature maps are produced, they are combined into a saliency map. However, since cell images have a higher contrast for color than for orientation and saturation, as distinct from nature scenes, different weights need to be applied to each feature map when they are combined into a saliency map. In the present study, the most accurate Attention Windows were produced when the color weight was 0.6, the orientation weight was 0.2, and the saturation weight was 0.2. Finally, the three feature maps are normalized and summed into a single saliency map using their weights and Eq. (4). After generating the saliency map *C_m_*, it is up-sampled to the original size.

(4) $$C_{m}=w_{1}\cdot \bar{C}(x,y)+w_{2}\cdot \bar{L}(x,y)+w_{3}\cdot \bar{O}(x,y)$$

### Saliency strength map generation

**Figure 5:**
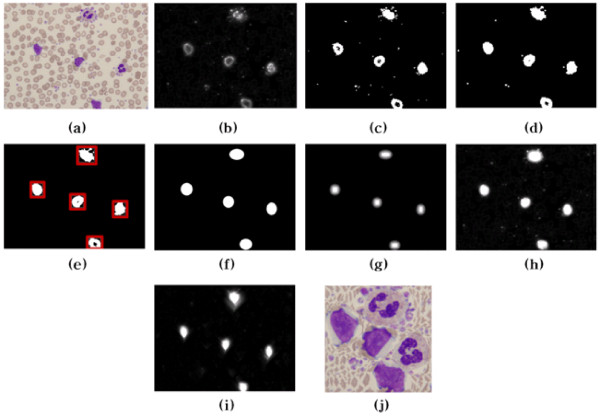
**Saliency strength using EAWs and energy map**: (a) original image (b) saliency map, (c) optimal thresholding, (d) morphological opening, (e) removal of small regions, (f) EAWs, (g) strength of EAWs, (h) Saliency Strength Map, (i) final energy map of (h), and (j) resized image.

Based on the saliency map, Attention Windows (AWs) are then detected to remove useless regions from the image, such as red blood cells and background, thereby improving the quality of the resized image. To determine the proper location of the AWs, optimal thresholding [16] of the saliency map is performed first (Fig. 5-(c)). This thresholding method is known to produce the best performance when an image only contains two principal regions (e.g. objects and background) and the distribution of the gray-level values in each region follows a Gaussian distribution [16]. Morphological closing is then performed to fill the holes in the nuclei of the WBCs (Fig. 5-(d)). Using the resulting binary image, region labeling is performed and small regions are considered as noise and removed (Fig. 5-(e)). At the same time, the initial AW positions (x, y) for each remaining region are estimated by X-Y projection. Also, since WBCs tend to have a round shape, the elliptical shapes of the AWs (EAWs) are re-estimated using a centroid and the radius of the initial AW (Fig. 5-(f)). Thereafter, a distance transform using the Euclidean distance is performed to boost the intensity difference between the central and boundary regions, termed the strength of the EAW (*EAW_S_*). As shown in Fig. 5-(g), the intensity strength of the boundary is lower than that of the central region in each nucleus.

The six steps for extracting the AW and estimating the *EAW_S _*are as follows:

***Step1: ***Otsu [17]'s optimal threshold *t_op _*is applied to the saliency map (*C_m_*).

(5) $$I(x,y)=\{\begin{array}{cc} 1 & if\;C_{m}(x,y)>t_{op}\\ 0 & otherwise \end{array}$$

***Step2: ***Morphological opening is performed to fill any holes in the cell nuclei.

***Step3: ***After region labeling, small regions are removed if the size of a region is below a predefined minimum threshold (3% of all image pixels). This predefined minimum threshold was determined by analyzing the minimum cell region from whole training cell regions.

***Step4: ***The initial position of the AW in each region is estimated using an X-Y projection.

***Step5: ***The elliptical AWs (EAW) are re-estimated using the centroid and radius of the initial AW.

***Step6: ***A distance transform is performed and the strength of the EAW(*EAW_S_*) estimated.

The saliency map and strength of the EAWs are then summed into a single saliency strength map (*SSM*) and normalized into 0~255, as shown in Fig. 5-(h).

(6) $$SSM(x,y)=g(\frac{1}{2}\cdot [C_{m}(x,y)+EAW_{S}(x,y)])$$

where g represents a Gaussian smoothing operator to reduce minor noise.

Finally, the resized image based on the SSM and its energy map is shown in Fig. 5-(j).

### Seam removal using energy map based on saliency strength map

**Figure 6:**
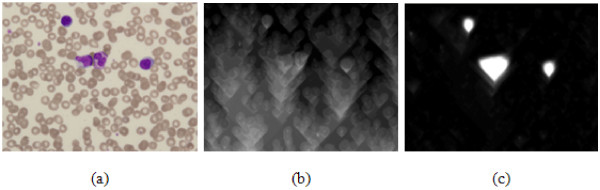
**Energy map comparison**: (a) original image (b) energy map using only gradient magnitude, and (c) energy map using saliency strength of SSM.

A seam is a monotonic and connected path of pixels proceeding from the top of an image to the bottom, or from left to right. Thus, when a seam is removed from an image, the image size is reduced by one in either the horizontal or vertical dimension. Likewise, seam carving uses an energy function defined based on the pixels to successively remove the minimum energy paths from an image [17]. Yet, as shown in 6-(b), an energy map using only the gradient magnitude introduces visual artifacts, regardless of the importance of the cells. Whereas the proposed energy maps in Figs. 5-(i) and 6-(c) show only the highest energy, indicating the existence of WBCs without visual artifacts.

Seam carving uses two types of energy removal strategy: backward and forward. Backward energy strategies are based on evaluating the energy, yet they introduce visual artifacts due to their seam removal strategy. The seams containing the lowest energy are removed one after another, however, the energy inserted into the new edges created by previously non-adjacent pixels that become new neighbors is ignored after a seam is removed. Thus, to reduce these visual artifacts, forward energy strategies [17] substitute an energy evaluation that calculates three possible seam step costs and defines the minimal amount of energy inserted by the removal of a seam.

In the seam removal procedure used in this study, the saliency strength of the SSM is used to correspond to the computing energy for each pixel. Computing the cost of the saliency strength then produces the same result as the forward energy strategy of Rubinstein et al. [17]. As distinct from backward energy strategies, three possible cases are then calculated to remove seams with forward energy strategies using the pixel values of *SSM(i, j) *and the following formula:

(7) $$\begin{array}{l} \left(a)\;C_{L}(i,j)=|SSM(i,j+1)-SSM(i,j-1)|+|SSM(i-1,j)-SSM(i,j-1)\right|\\ \left(b)\;C_{U}(i,j)=|SSM(i,j+1)-SSM(i,j-1)\right|\\ \left(c)\;C_{R}(i,j)=|SSM(i,j+1)-SSM(i,j-1)|+|SSM(i-1,j)-SSM(i,j+1)\right| \end{array}$$

where *SSM*(*i*, *j*-1) is the new pixel that is replaced after removing *SSM*(*i*, *j*), *SSM*(*i*, *j*+1) and *SSM*(*i*-1, *j*) are the new right and upper neighbors, respectively, and *C_L_*, *C_U_*, and *CR *represent the costs of the three possible vertical seams.

A cost matrix M is then created to compute the seams.

(8) $$M(i,j)=P(i,j)+\min\{\begin{array}{l} M(i-1,j-1)+C_{L}(i,j)\\ M(i-1,j)+C_{U}(i,j)\;\;\\ M(i-1,j+1)+C_{R}(i,j) \end{array}$$

where *P*(*i*, *j*)is the gradient value obtained from *SSM(i, j)*, *M*(*i*-1, *j*-1) is the left upper neighbor, and *C_L _*is its cost. The cost of the corresponding upper *M*(*i*-1, *j*) neighbors *C_U _*and right upper *M*(*i*-1, *j*+1) neighbors *C_R _*are computed in the same manner to determine the minimum energy of the new saliency strength after removing *SSM*(*i*, *j*).

Once the cost matrix is constructed, *M*(*i*, *j*) in a random position represents a pixel (*i*, *j*) in a path crossing the image from top to bottom, and is connected to other adjacent pixels containing the minimal energy according to the saliency strength. Consequently, the resizing is performed by iteratively creating a cost matrix after blending the gaps arising from seam removal.

**Figure 7:**
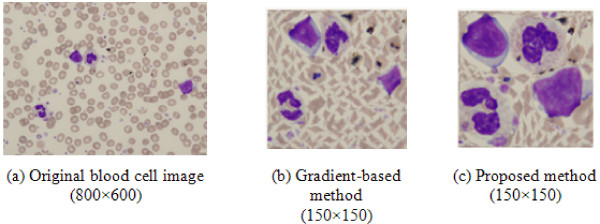
**Comparison of seam carving results**: (b) resized image using original seam carving and (c) resized image using proposed method.

Fig. 7 shows a comparison of the results of seam carving when using a gradient-based energy map and the proposed energy map. While the gradient-based energy map distorts the WBC nuclei due to a harsh reduction of the image size, the proposed method is able to preserve the original shape of the WBC nuclei.

## Results and discussion

The experimental tests used color peripheral blood images collected at the Severance Hospital, Yonsei University. The 8 test images were based on a slide of a peripheral blood smear and taken using a microscope, charge-coupled device (CCD) camera, and 24-bit digitizer with an 800 × 600 image size.

As there is no specific method for evaluating the performance of image resizing, the Peak Signal-to-Noise Ratio (PSNR) was used first to evaluate the content preservation of the proposed method. Plus, the Ratio of Distortion (ROD) was applied to evaluate the geometric distortion of the resized images. Note that, the size of the source images was 800 × 600 and the target size was automatically determined according to the size of the EAWs to include all the nuclei without distortion.

### PSNR (Peak Signal-to-Noise Ratio) comparison

An experimental comparison of seam carving is generally very difficult as there are no standard criteria for performance tests. Thus, to validate the effectiveness of the proposed approach, this study used the Peak Signal-to-Noise Ratio (PSNR).

The PSNR originally comes from electronics and represents the ratio between the maximum possible power of a signal and the power of the noise that affects the fidelity of its representation. However, in the field of image processing, the PSNR is used to measure of the quality of an image or its compression. In the case of image processing, the maximum value (*MAX*) of intensity level 255 is used instead of the maximum possible power. In Eq. (9), the MSE represents the mean square error between the original image *I(i, j) *and the target image *K(i, j)*.

(9) $$MSE=\frac{1}{mn}\sum_{i=0}^{m-1}\sum_{j=0}^{n-1}||I(i,j)-K(i,j)|{|}^{2}$$

Where *m *and *n *represent width and height of the image, respectively.

The PSNR is then estimated by computing the ratio of the maximum value to the MSE using Eq. (10).

(10) $$PSNR=10\cdot \log_{10}\left(\frac{MAX_{I}^{2}}{MSE}\right)=20\cdot \log_{10}\left(\frac{MAX_{I}}{\sqrt{MSE}}\right)$$

For the performance test, gradient-based seam carving and the proposed method were applied to the source images to create target images. The WBC nuclei were then cropped manually from each image with a graphic tool and used for the PSNR comparison.

**Figure 8:**
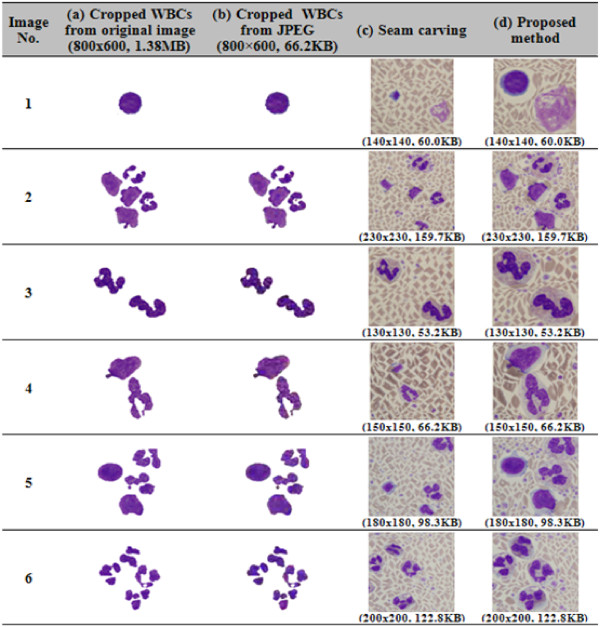
**Results of image quality when using JPEG compression method, seam carving, and proposed method**.

Fig. 8 shows the image quality results when using a JPEG compression method, image resizing with seam carving, and image resizing with the proposed method. Clearly, the seam carving based on the gradient energy produced WBC nuclei with distorted shapes and sizes, whereas the JPEG compression and the proposed method preserved the shapes and sizes of the WBC nuclei.

**Figure 9:**
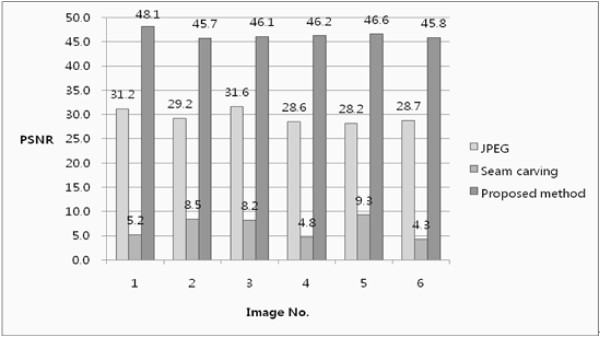
**PSNR comparison of JPEG compression, seam carving, and proposed method**.

In addition, Fig. 9 shows a comparison of the PSNR results for the three methods. In this case, since the seam carving based on the gradient energy produced severe distortion and was unable to preserve the ROIs accurately, it had a very low PSNR ratio. Meanwhile, the JPEG compression had a high average PSNR ratio at 29.6. However, the proposed method produced an average PSNR ratio of 46.4, which was very close to the original image quality.

Thus, although JPEG compression can produce the perception of identical results with the original WBCs, there is a loss of image quality, as shown in Figs. 8-(b) and 9, meaning the characteristics needed for WBC segmentation and classification are not always preserved. In contrast, since the proposed method is able to preserve the original image quality, better segmentation and classification results can be expected in the post-processing steps. Therefore, the proposed image resizing algorithm can be very useful for reducing the cell analysis and memory storage in the case of medical images, especially blood cell images.

### ROD (Ratio of Distortion) comparison

To evaluate the ratio of distortion (ROD) of the WBC nuclei after image resizing, a new evaluation method was used based on the geometric properties of the objects. First, three different users were asked to crop the WBC nuclei from the resized images shown in Figs. 8(c) and 8(d) using a graphic tool, and only those WBCs where at least two users were in agreement were then used for the comparison.

**Figure 10:**
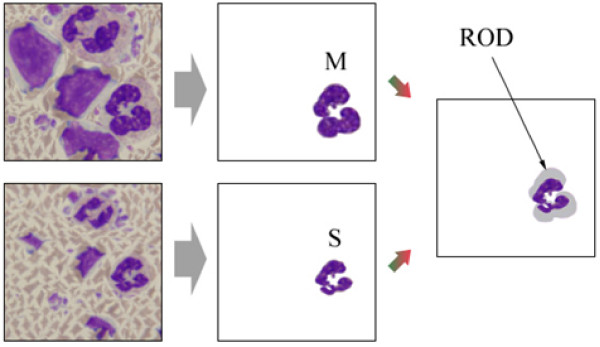
**ROD (Region of Distortion) evaluation criteria for image resizing performance**.

The distortion results for each method were compared with the manually cropped WBC nuclei and the error ratio estimated using Eq. (11).

(11) $$ROD=\frac{Card(M-(M\cap S))}{M}$$

where M represents the set of pixels in the WBC nuclei in the original image and S represents the set of pixels in the WBC nuclei in the resized image when using seam carving and the proposed method. The symbol Card (A) denotes the cardinality of set A. Fig. 10 shows the ROD errors marked by arrows for the automatically extracted WBCs when compared with the manually selected WBCs.

Table 1 shows the performance evaluation results when using Eq. (11), where the proposed method showed a lower average ROD ratio of 0 when compared to that for the seam carving at 0.65.

**Table 1:** ROD comparison of seam carving and proposed method

**Image No**.	Seam carving	Proposed method
1	0.89	0

2	0.53	0

3	0.47	0

4	0.89	0

5	0.59	0

6	0.58	0

The seam carving results produced distortions of 0.5~0.8, whereas no distortion occurred with the proposed method. As such, the seam carving was unable to preserve the WBCs when the image was resized harshly. Since an energy map using the original gradient magnitude of the WBCs is not distinctive in blood images, this makes it hard to apply a seam carving operator to blood cell images, and the WBC contents are inevitably affected during the pixel removal. In contrast, the proposed method was able to preserve the WBCs exactly, as it used the SSM to maximize the energies of the WBCs and minimize the energies of the red blood cells and background.

## Conclusions

This paper proposed a new image compression method that uses a Saliency Strength Map (SSM) and seam carving to preserve important contents, such as WBCs included in blood cell images, with a lower file size. The SSM is constructed using a visual attention model and the structural properties of WBCs to generate a new energy map. Thus, the purpose of the map is to maximize the energies of the WBCs, while minimizing the energies of the red blood cells and background.

In experiments, the proposed method was shown to improve the file compression performance when compared to JPEG. Nonetheless, despite the improved performance of seam carving based on an SSM, additional computation time is required depending on the image resolution. Therefore, a faster cell-image resizing method is currently being investigated to reduce the computation time, while maintaining the same image quality.

## Competing interests

The authors declare that they have no competing interests.

## Authors' contributions

BCK designed the algorithm and wrote the paper; SHK implemented and tested the algorithm according to the advice of BCK; JYN was involved in various discussions and gave suggestions for a flawless system; All the authors read and approved the final manuscript.
